# A Transfer Learning Radiomics Nomogram for Preoperative Prediction of Borrmann Type IV Gastric Cancer From Primary Gastric Lymphoma

**DOI:** 10.3389/fonc.2021.802205

**Published:** 2022-01-11

**Authors:** Bao Feng, Liebin Huang, Yu Liu, Yehang Chen, Haoyang Zhou, Tianyou Yu, Huimin Xue, Qinxian Chen, Tao Zhou, Qionglian Kuang, Zhiqi Yang, Xiangguang Chen, Xiaofeng Chen, Zhenpeng Peng, Wansheng Long

**Affiliations:** ^1^ Department of Radiology, Affiliated Jiangmen Hospital of Sun Yat-Sen University, Jiangmen, China; ^2^ School of Electronic Information and Automation, Guilin University of Aerospace Technology, Guilin, China; ^3^ School of Automation Science and Engineering, South China University of Technology, Guangzhou, China; ^4^ Department of Radiology, Meizhou People’s Hospital, Meizhou, China; ^5^ Department of Radiology, The First Affiliated Hospital of Sun Yat−Sen University, Guangzhou, China

**Keywords:** Borrmann type IV gastric cancer, primary gastric lymphoma, transfer learning, whole slide image, deep learning

## Abstract

**Objective:**

This study aims to differentiate preoperative Borrmann type IV gastric cancer (GC) from primary gastric lymphoma (PGL) by transfer learning radiomics nomogram (TLRN) with whole slide images of GC as source domain data.

**Materials and Methods:**

This study retrospectively enrolled 438 patients with histopathologic diagnoses of Borrmann type IV GC and PGL. They received CT examinations from three hospitals. Quantitative transfer learning features were extracted by the proposed transfer learning radiopathomic network and used to construct transfer learning radiomics signatures (TLRS). A TLRN, which integrates TLRS, clinical factors, and CT subjective findings, was developed by multivariate logistic regression. The diagnostic TLRN performance was assessed by clinical usefulness in the independent validation set.

**Results:**

The TLRN was built by TLRS and a high enhanced serosa sign, which showed good agreement by the calibration curve. The TLRN performance was superior to the clinical model and TLRS. Its areas under the curve (AUC) were 0.958 (95% confidence interval [CI], 0.883–0.991), 0.867 (95% CI, 0.794–0.922), and 0.921 (95% CI, 0.860–0.960) in the internal and two external validation cohorts, respectively. Decision curve analysis (DCA) showed that the TLRN was better than any other model. TLRN has potential generalization ability, as shown in the stratification analysis.

**Conclusions:**

The proposed TLRN based on gastric WSIs may help preoperatively differentiate PGL from Borrmann type IV GC.

Borrmann type IV gastric cancer, primary gastric lymphoma, transfer learning, whole slide image, deep learning.

## Introduction

Gastric cancer (GC) and primary gastric lymphoma (PGL) are the two most commonly encountered gastric malignancies (1), whose treatment strategies are different (2). Surgical resection remains the main treatment option for GC, especially for patients who may be cured by radical resection. However, the best PGL treatment options are chemotherapy or radiotherapy. Therefore, accurate GC and PGL differentiation before treatment is critical to choosing treatment options to avoid unnecessary surgery in patients with PGL.

Endoscopy biopsies are generally used to diagnose Borrmann type IV GC and PGL because of their high sensitivity. However, previous studies showed that biopsy testing might not accurately locate lesions, leading to high false-negative rates (3–5). Moreover, the biopsy is an invasive procedure that may increase potential perforation risk (4).

Noninvasive computed tomography (CT), widely used for differential and preoperative diagnoses, therapeutic evaluation, and staging in patients with gastric malignancies, can help find tumor lesions (5, 6). Studies have shown that CT subjective findings (e.g., gastric wall thickness and enhancement pattern) play an important role in diagnosing gastric malignancies (7). However, distinguishing Borrmann type IV GC and PGL in lesion distribution, irregular gastric wall thickness and enhancement pattern, and so on is difficult with CT (8). Thus, an effective method is needed to differentiate Borrmann type IV GC and PGL.

Convolutional neural networks (CNN) and other deep learning models have shown great potential in medical imaging (e.g., bladder cancer treatment (9), preoperative meningioma grading (10), individual induction chemotherapy in advanced nasopharyngeal carcinoma (11), and so on). CNN can increasingly learn high-order features from images of large neural networks and extract valuable features to the desired outputs (12). However, the training dataset size is crucial to building a robust deep learning model. Obtaining a large number of medical images is difficult in clinical practice (13). Thus, developing a method to improve the deep learning model’s performance for the small dataset is necessary.

The use of transfer learning is ubiquitous to ameliorate the dataset effect (14). Transfer learning (TL) improves model performance in target tasks by transferring features from source tasks that have already been learned (15, 16). Moreover, TL has been gradually applied in recent years to many medical image analytical fields (e.g., image segmentation, lesion localization, and lesion pattern recognition) (17, 18). However, a key factor in the performance of TL strategy is the similarity between the source and target domain dataset (19, 20). From the present strategies studied, using the natural image datasets (e.g., ImageNet dataset) as the source domain dataset for pretraining in transfer learning-based methods is common (21). However, natural images showed a weak correlation with medical images, which may decrease model performance or even cause a negative transformation (20). Medical gastric images (e.g., gastroscopic images, CT images, and whole slide images (WSIs), and so on), which contained different information and reflected the tumor information of local lesions from a different level, could be more relevant to the task. Thus, this study developed a TL model, pre-trained with the WSIs of GC as the source domain dataset, and fine-tuned the model on CT images. The TL model, combining macroscopical and microcosmic information of tumors, might provide prospects for constructing a more powerful model to distinguish between Borrmann type IV GC and PGL.

This retrospective study aims to design a reliable model that takes advantage of the more combined potential information to differentiate Borrmann type IV GC and PGL preoperatively. This tool will help identify patients that need active treatment.

## Materials And Methods

### Patients

#### Internal Dataset

The hospital’s institutional review board approved this retrospective study and waived informed consent. The basic inclusion and exclusion criteria for the training and validation sets are shown in **Supplementary A1**. Moreover, 184 patients pathologically diagnosed as Borrmann type IV GC and PGL were divided into the training (*n* = 110, January 2017–March 2018) and internal validation (*n* = 74, April 2018–December 2019) cohorts in a 6:4 ratio.

#### External Dataset

Data from patients with pathologically proven Borrmann type IV GC or PGL, who had undergone CT from two other independent institutions (the First Affiliated Hospital of Sun Yat-sen University [*n* = 123] and Meizhou People’s Hospital [*n* = 131]), were also approved by the institutional research ethics board where the data originated. The exclusion criteria were similar to the internal dataset.

#### The Pathological Dataset

**Figure 1:**
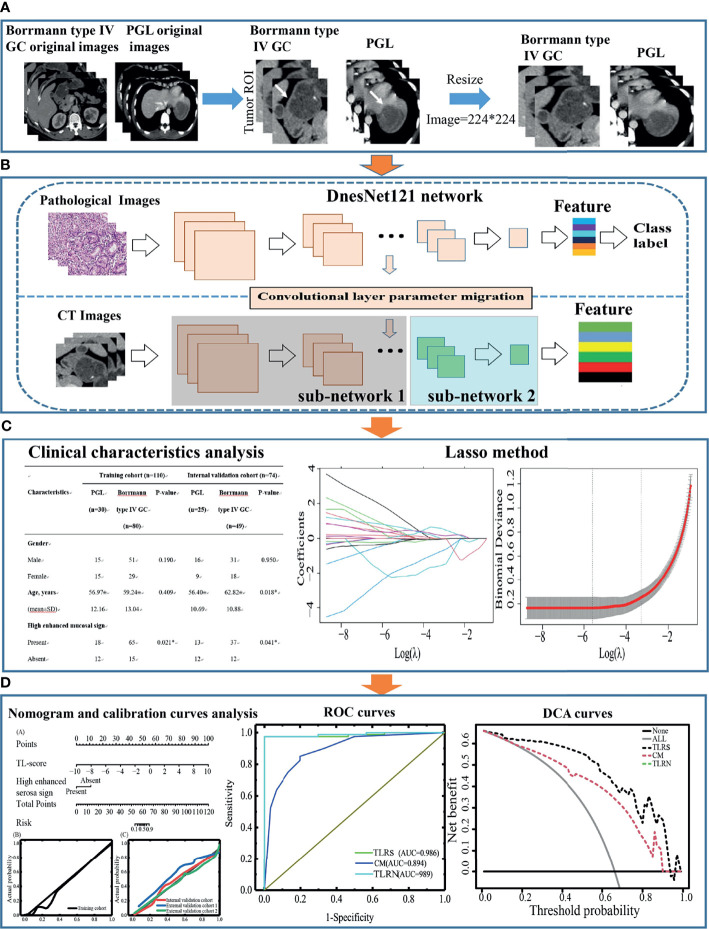
The design flow chart of this paper. **(A)** Acquisition of ROI images for Borrmann type IV GC and PGL. **(B)** The ROI features extraction by Densenet121. **(C)** Transfer learning features selection process and model building, statistical analysis of clinical characteristics, and CT subjective findings. **(D)** Performance evaluation of the transfer learning radiomics nomogram. GC, gastric cancer; PGL, primary gastric lymphoma; ROI, region of interest.

The pathological dataset, which contained 1,730 WSIs of gastric cancer from the Mars Data Science Platform (https://www.marsbigdata.com/), was used as the source domain of TL. **Figure 1** shows the design flow chart of this paper.

### Pathological And CT Subjective Findings Evaluation

All the surgical or biopsy specimens of the patients were taken in 3-mm serial sections and stained with hematoxylin and eosin. Two pathologists evaluated the specimens (pathologists 1 and 2 with 10 and 15 years of experience, respectively, in the pathological diagnosis of GC and PGL). The Borrmann classification was confirmed by the GC Japanese classification (22). The PGL diagnosis was assessed by the fourth edition of the WHO Classification of Tumors of Haematopoietic and Lymphoid tissues (23). Two radiologists (readers 1 and 2 with 10 and 15 years, respectively, of experience reviewing abdominal CT images) were blinded to the clinical information and histopathologic diagnosis. A consensus was reached by discussion in case of disagreement. **Supplementary A2** shows the CT subjective findings evaluation in detail.

### Images Acquisition And Segmentation

All of the patients fasted for at least 5 h and were encouraged to drink 600–1,000 mL of water 30 min before CT examination. All CT scans were performed with the patient in the supine position with the entire abdominal area under deep breathing. **Supplementary A3** shows the details of the CT protocol.

For the arterial and portal venous contrast-enhanced CT images on the axial plane, the region of interest (ROI) for the Borrmann type IV GC and PGL refers to their tumor lesion areas for quantitative analysis. **Supplementary A4** shows the pretreatment images.

### TL Radiopathomic Network And Signature Construction

**Figure 2:**
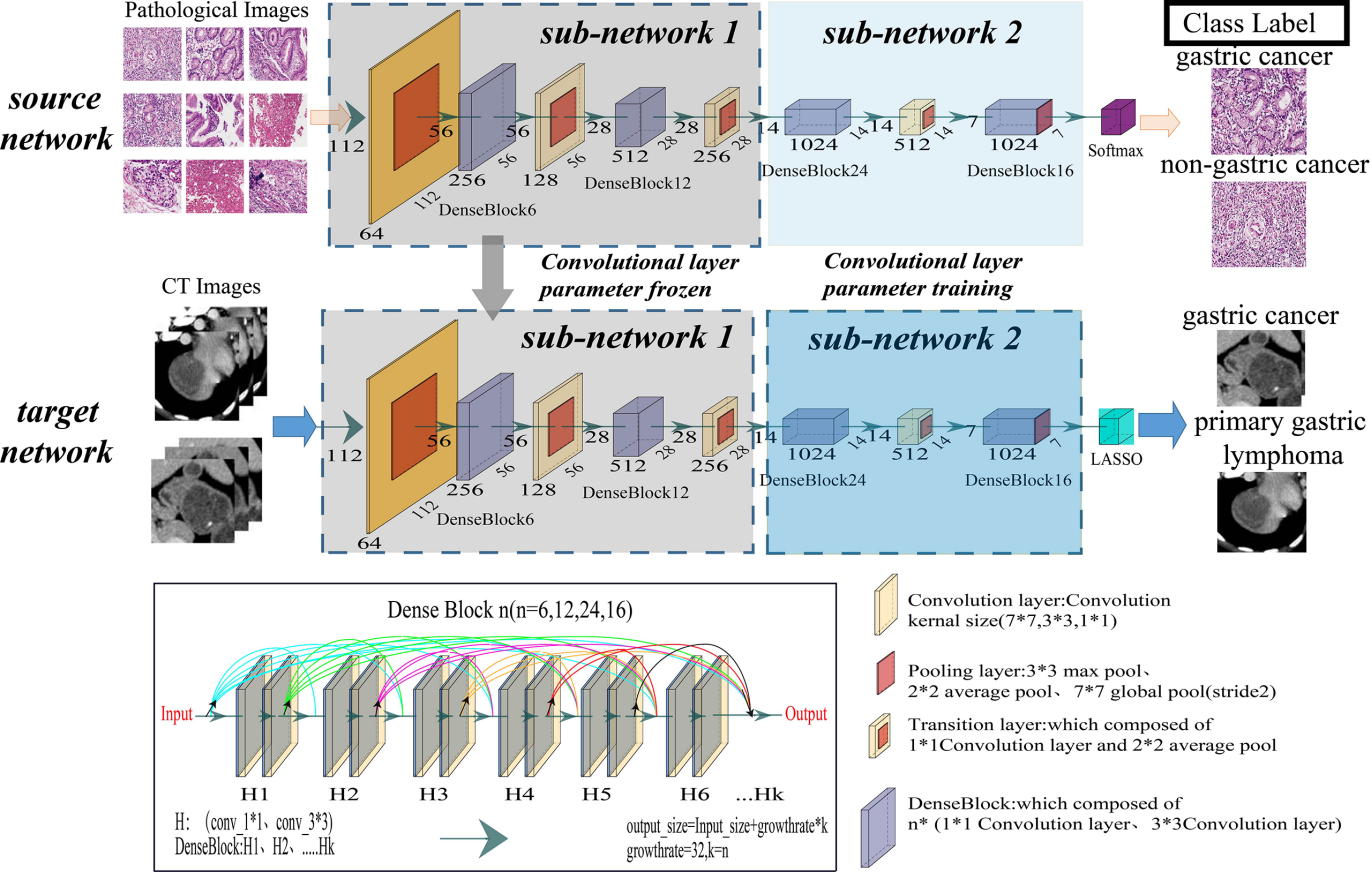
Illustration of the overall transfer learning framework of this study. The convolutional layer of the DenseNet121 model was taken out as the feature extractor of this study.

TL (14–18) migrates a network trained on a large dataset to a different related task and avoids overfitting problems caused by insufficient training data in regular deep learning. This study proposed a transfer learning radiopathomic network (TLRPN), which consisted of two parts (parts 1 and 2). Firstly, all parameters of the DenseNet121 network were trained by the WSIs of GC, and the parameters of the first 39 convolutional layers were frozen (part 1). Secondly, part 2 included the other convolutional layers trained by the dataset of CT images. Moreover, the parameters of the other network layers were fine-tuned because these become progressively more specific to the subtle features. **Figure 2** describes the proposed TL framework. The experimental details are provided in the **Supplementary A5**.

Based on the TLRPN, 11,264 transfer learning features were extracted, and the classification layer was trained. The details of TL feature extraction and selection are presented in **Supplementary A5**. In the classification layer training step, the least absolute shrinkage and selection operator (LASSO) logistic regression was used to build the transfer learning radiomics signature (TLRS), built by linearly combining the LASSO-selected features with their corresponding coefficients. Finally, the features with non-zero coefficients were considered valuable predictors for predicting Borrmann type IV GC and PGL.

### Visualization Of The Transfer Learning Radiomics Signature

To further understand TLRS, the visualization algorithms were used to display how the network learned the tumor features (24). Generally, TLRS mainly visualizes the transfer learning features learned by the convolutional layer and the output response of each lesion corresponding to the convolutional layer. The visualized TLRS process was developed in three steps. Firstly, the ROI images were regarded as the TLRPN input. Secondly, the filter visualization technique was used to visualize each convolution filter response situation on these ROI images from the different lesion types. Finally, diagnostic feature maps were visualized, which contributed to understanding the conventional filters and enabling a quick educated diagnosis.

### Development Of Transfer Learning Radiomics Nomogram

The transfer learning radiomics nomogram (TLRN), incorporating TLRS, clinical factors, and CT subjective findings, was built with multivariable logistic regression analysis by inputting variables found to be significant on univariate analysis. The backward stepwise selection method was used in which the likelihood ratio test of the Akaike information criterion was regarded as the stopping criterion.

### Independent Validation Of The TLRN

The area under the curve (AUC) of receiver operating characteristic curve (ROC) with 95% confidence interval (95% CI), sensitivity, specificity, accuracy, positive predictive value (PPV), and negative predictive value (NPV) (25) were to evaluate the performance of TLRN and the Delong test was used to compare different AUCs. The highest Youden’s index on ROC curve of the training set was regarded as optimal cut-off value, which used to differentiate the patients’ groups of the Borrmann type IV GC and PGL. To estimate the clinical usefulness of the TLRN, the decision curve analysis (DCA) was performed with net benefits for threshold probabilities.

### Statistical Analysis

The independent-samples t-test or the Mann–Whitney U-test was used for continuous variables. The Fisher’s exact test or chi-square test was used for categorical variables to assess the differences in patient characteristics between the two groups. All statistical analyses were two-tailed. A p value less than 0.05 was considered to be statistically significant. **Supplementary A6** presents the statistical software.

## Results

### Clinical Characteristics Analysis And Model Building

The clinical characteristics of the patients in the sets are listed in **Table 1-1** and **1-2**. There was significant difference in high enhanced mucosal sign, high enhanced serosa sign, nodular or an irregular outer layer of the gastric wall, and perigastric fat infiltration between Borrmann type IV GC and PGL in the training and validation sets (all *P* < 0.05). In addition, they were all significantly different between groups in the training and validation sets (all *P* < 0.05).

**Table 1-1:** Clinical characteristics of PGL and Borrmann type IV GC patients in the training and internal validation cohorts.

Characteristics	Training cohort (*n* = 110)			Internal validation cohort (*n* = 74)		
	PGL (*n* = 30)	Borrmann type IV GC(*n* = 80)	*P* value	PGL (*n* = 25)	Borrmann type IV GC (*n* = 49)	*P* value
**Gender**						
Male	15	51	0.190	16	31	0.950
Female	15	29		9	18	
**Age, years** (mean ± SD)	56.97 ± 12.16	59.24 ± 13.04	0.409	56.40 ± 10.69	62.82 ± 10.88	0.018*
**High enhanced mucosal sign**						
Present	18	65	0.021*	13	37	0.041*
Absent	12	15		12	12	
**High enhanced serosa sign**						
Present	4	62	<0.001*	1	30	0.001*
Absent	26	18		24	19	
**Nodular or an irregular outer layer of the gastric wall**						
Present	10	63	<0.001*	9	38	<0.001*
Absent	20	17		16	11	
**Perigastric fat infiltration**						
Present	8	61	0.001*	9	34	0.006*
Absent	22	19		16	15	
**TL score: median (interquartile range)**	1.425 (0.070 to 2.547)	−2.516(−4.183 to −1.821)	<0.001*	0.068 (−1.038 to 1.840)	−2.657 (−3.982 to −1.233)	<0.001*

**Table 1-2:** Clinical characteristics of PGL and Borrmann type IV GC patients in the external validation cohorts 1 and 2.

Characteristics	External validation cohort 1 (n = 123)			External validation cohort 2 (n = 131)		
	PGL (*n* = 48)	Borrmann type IV GC (*n* = 75)	*P* value	PGL (*n* = 50)	Borrmann type IV GC (*n* = 81)	*P* value
**Gender**						
Male	29	46	0.919	28	57	0.094
Female	19	29		22	24	
**Age, years** (mean ± SD)	63.05 ± 10.81	64.23 ± 12.12	0.370	55.25 ± 11.37	61.81 ± 10.33	0.531
**High enhanced mucosal sign**						
Present	25	36	0.659	29	40	0.337
Absent	23	39		21	41	
**High enhanced serosa sign**						
Present	7	26	0.014*	45	55	0.004*
Absent	41	49		5	26	
**Nodular or an irregular outer layer of the gastric wall**						
Present	28	49	0.434	15	67	<0.001*
Absent	20	26		35	14	
**Perigastric fat infiltration**						
Present	20	55	0.083	21	60	<0.001*
Absent	28	20		29	21	
**TL score: median (interquartile range)**	0.017 (0.001 to 0.038)	−0.1754 (−0.998 to −0.053)	<0.001*	−0.371 (−0.999 to −0.0261)	−0.023 (−0.060 to −0.001)	<0.001*

SD, standard deviation; PGL, primary gastric lymphoma; GC, gastric cancer; TL, transfer learning. *Statistically significant.

The clinical model (CM) incorporates four CT subjective findings as multivariable logistic regression inputs. However, high enhanced serosa sign, nodular or an irregular outer layer of the gastric wall, and perigastric fat infiltration were selected as independent predictors for CM. **Table S1** shows the results.

### Diagnostic TLRs Performance

In total, 1,998 TL features significantly differed by Mann–Whitney *U-*test between the Borrmann type IV GC and PGL groups. Then, 100 TL features were obtained by applying the criteria of minimum redundancy maximum relevance. Among these, 10 TL features with non-zero coefficients were selected by LASSO logistic regression, which were used to develop a TL score calculation formula (**Supplementary A7**).

### TLRN Construction And Validation

On multivariate analysis, high enhanced serosa sign (*P* = 0.026; OR, 0.046; 95% CI, 0.003–0.697) and TL score (*P* < 0.001; OR, 0.462; 95% CI, 0.322–0.661) were identified as independent indicators to TLRN building (**Table S2**). By incorporating these independent factors, a combined model was constructed and presented as a radiomics nomogram (**Figure 4A**). Using the calibration curve, a marked connection between the predicted and actual data in the training and validation cohorts was confirmed (**Figure 4B, C** **)**.

### Method Evaluation And Comparison

#### Performance Comparison Between Transfer and Nontransfer Learning

In this position, a nontransfer learning radiomics signature (NTLRS), based on the DenseNet121 network, was developed. The TLRS performance was analyzed based on the pathological image of GC and NTLRS. In the internal validation and two external cohorts, the under the curves (AUCs) of TLRS were 0.904 (95% CI, 0.814–0.961), 0.834 (95% CI, 0.756–0.895), and 0.894 (95% CI, 0.828–0.941), which were 0.076, 0.097, and 0.031 higher than the AUCs of NTLRS, respectively. The ROC curve is shown in **Figure S4A**.

#### Performance Comparison Between TLRS and Transfer Learning Based on Source Data 1: ImageNet Dataset

A transfer learning signature was developed based on the ImageNet datasets (named the TLRS-ImageNet) to analyze the impact of source domain data for transfer learning. **Figure S4B** showed the ROC curves of the prediction results for the two models. For the results, AUCs of TLRS reached 0.904 (95% CI, 0.814–0.961), 0.834 (95% CI, 0.756–0.895), and 0.894 (95% CI, 0.828–0.941), which were significantly better than the TLRS-ImageNet. Compared with the TLRS-ImageNet, these results indicated that the TLRS made critical contributions to improve accuracy.

#### Performance Comparison Between TLRS and Transfer Learning Based on Source Data 2: Pathological Images of the Lung

In addition, the transfer learning radiomics signature (named TLRS-Lung), which was pre-trained by pathological images of lung cancer from the TCGA, was developed. Compared with the TLRS, the TLRS-Lung showed unsatisfying performances in distinguishing between PGL and Borrmann type IV GC in the internal cohort (AUC = 0.774), external validation cohort 1 (AUC = 0.787), and external validation cohort 2 (AUC = 0.821). **Figure S4C** shows the details of the result.

#### Performance Comparison Between VGG16, Extreme Learning Machine, Hand-Crafted Radiomics Signature, and TLRS

To comprehensively evaluate the TLRS performance, the TLRS was compared with the other three methods: (1) the hand-crafted radiomics signature (HCRS) was developed by the following steps: ROI acquirement, feature extraction, feature selection, and model construction (**Supplementary A8**); (2) the VGG16 network (26); and (3) the extreme learning machine (ELM). The results showed that the TLRS indicated higher AUCs than the other three methods. **Table S3** shows the diagnostic performance of the TLRS, HCRS VGG16, and ELM.

#### Performance Comparison Between CM, TLRS, and TLRN

**Figure 3:**
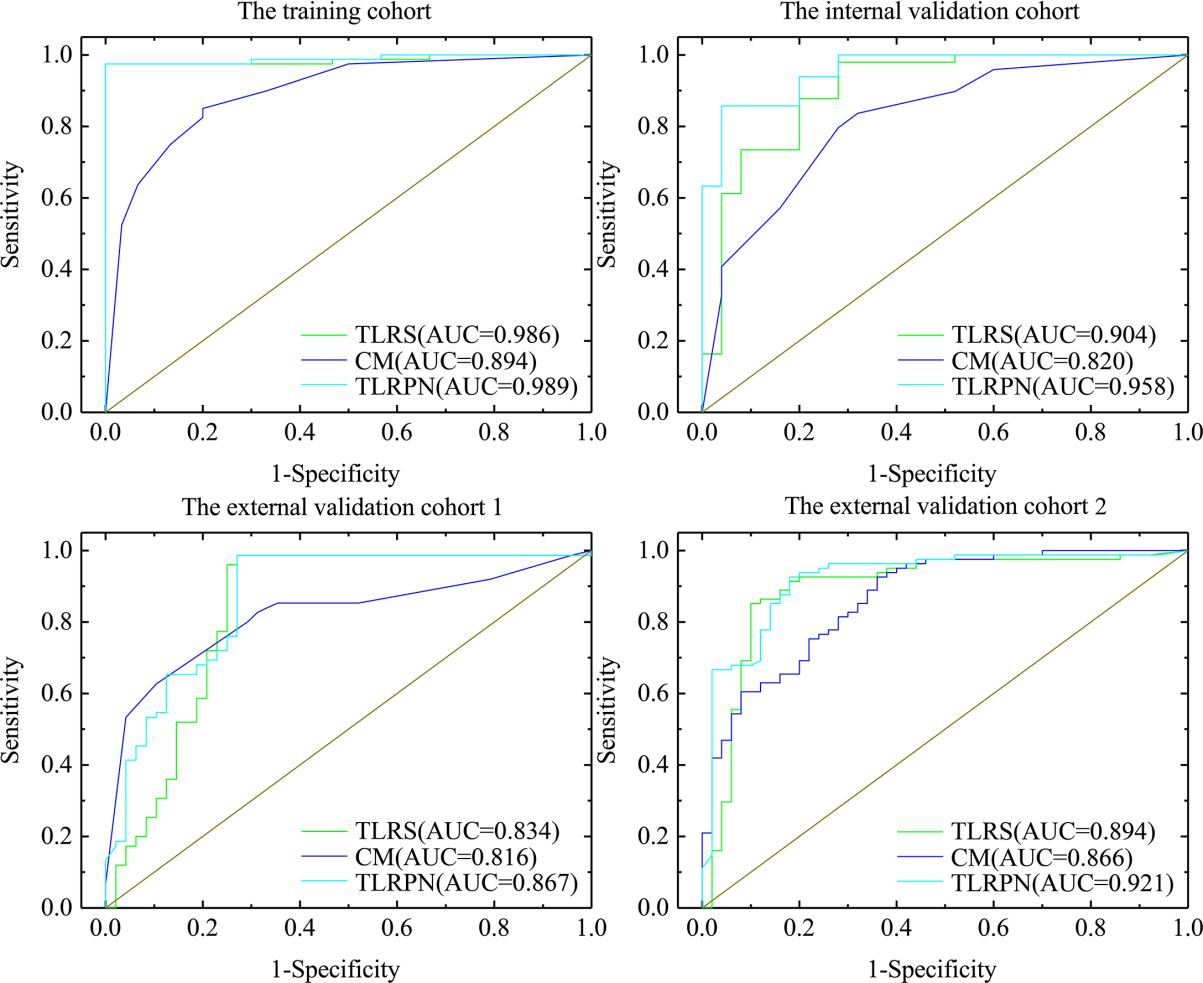
The AUCs of three model. The clinical model, transfer learning radiomics signature based on the pathological image of gastric cancer (TLRS) and transfer learning radiomics nomogram (TLRN).

The diagnostic performance of CM, TLRS, and TLRN are summarized in **Table 2** and **Figure 3**. For the internal and two external validation cohorts, CM showed lower AUC of 0.820 (95% CI, 0.718–0.921), 0.816 (95% CI, 0.736–0.880), and 0.866 (95% CI, 0.795–0.919), while the TLRN achieved the highest AUC of 0.958 (95% CI, 0.883–0.991), 0.867 (95% CI, 0.794–0.922), and 0.921 (95% CI, 0.860–0.961).

**Table 2:** Diagnostic performance of the HCR, CM, TLRS, and TLRN in the training and validation sets.

Model		AUC(95% CI)	Sensitive	Specificity	Accuracy	PPV	NPV
**Training cohort**	CM	0.894 (0.827–0.960)	0.850	0.800	0.836	0.919	0.667
	TLRS	0.986 (0.942–0.999)	0.975	0.967	0.973	0.987	0.936
	TLRN	0.989 (0.947–1.000)	0.975	0.967	0.973	0.987	0.936
**Internal validation cohort**	CM	0.820 (0.718–0.921)	0.720	0.796	0.770	0.936	0.667
	TLRS	0.904 (0.814–0.961)	0.980	0.720	0.892	0.872	0.947
	TLRN	0.958 (0.883–0.991)	0.857	0.960	0.891	0.976	0.774
**External validation cohort 1**	CM	0.816 (0.736–0.880)	0.827	0.687	0.772	0.805	0.717
	TLRS	0.834 (0.756–0.895)	0.986	0.729	0.886	0.850	0.972
	TLRN	0.867 (0.794–0.922)	0.987	0.729	0.886	0.851	0.972
**External validation cohort 2**	CM	0.866 (0.795–0.919)	0.926	0.640	0.817	0.807	0.842
	TLRS	0.894 (0.828–0.941)	0.852	0.900	0.872	0.932	0.790
	TLRN	0.921 (0.860–0.961)	0.926	0.820	0.886	0.893	0.872

AUC, area under curve; CI, confidence interval; CM, clinical model; TLRS, transfer learning radiomics signature; TLRN, transfer learning radiomics nomogram.

**Figure 4:**
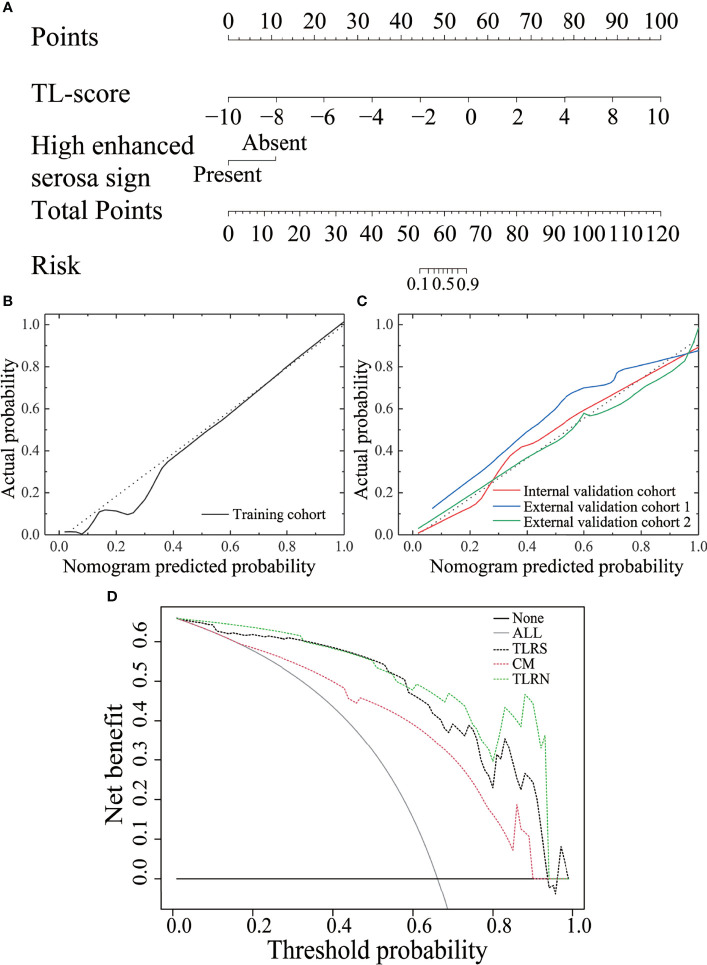
The performance of the transfer learning radiomics nomogram and curve analysis for the various models. **(A)** The TLRN is based on TLRS and CT subjective findings. Calibration curves of the TLRN in the training cohort **(B)** and three validation cohorts **(C)**. **(D)** Decision curve analysis for various models.

DCA indicated a higher net benefit for the TLRN in differentiating the GC and PGL groups than the clinical model. The threshold probability was within the range of 0.01 to 0.93 (**Figure 4D**).

## Discussion

The accurate and noninvasive PGL and Borrmann type IV GC classification is of crucial importance in clinical practice. Integrating lesion information on a more visual scale to develop a more reliable and generalized method to differentiate PGL and GC remains a challenging issue. This multicenter retrospective study built a preoperative TLRN using WSIs of GC and CT images with a transfer learning strategy to differentiate patients with PGL and Borrmann type IV GC. The proposed TLRN, combining useful lesion information from WSIs and CT images by using transfer learning strategy, showed better predictive performance in the internal validation cohort (AUC = 0.958), external cohort 1 (AUC = 0.867), and external cohort 2 (AUC = 0.921). The performance of the TLRN model indicated a strong association between high-dimensional CT and pathological images features. Therefore, the model served as a novel tool to accurately identify patients with Borrmann type IV GC and PGL and guide the treatment.

Subjective CT findings are the commonly used diagnostic and differential bases, which reflect the pathological characteristics of advanced GC and PGL to a certain extent. A high enhanced serosa sign was an important risk factor incorporated into the TLRN of the current study. Moreover, studies have shown that high enhanced serosa sign was a useful important factor for GC diagnosis (27). The high enhanced serosa sign by subjective CT findings evaluation was perfect, demonstrating that it was a robust subjective CT finding. In addition, the high enhanced serosa sign may be related to scirrhous metastasis in GC. When the GC tumor cells infiltrate all layers, it expands the mucosa, submucosa, and serosa rather than the muscularis propria because the muscle cells of muscularis propria are arranged more closely. Therefore, in a contrast-enhanced scan, the muscularis propria showed slightly low density due to poor enhancement. In contrast, the subserosal tissue and serosa showed obvious enhancement due to tumor invasion, expansion, and thickening, forming the high enhanced serosa sign (27).

The PGL originated from the submucosa layer, mainly diffusing infiltration growth. Moreover, tumor cell infiltration in the muscularis propria is relatively homogeneous compared with the other layers. The PGL density is then relatively homogeneous on enhanced CT images. Thus, the high enhanced serosa sign rarely appears in PGL (28). However, imaging data alone requires high diagnostic experience for radiographers because doctors’ subjectivity is too strong, large differences between observers exist, and subjective errors are also inevitable. Therefore, a method to compensate for the lack of clinical diagnostic methods and improve preoperative differentiation of PGL and Borrmann type IV GC is urgently needed.

This study used the deep learning method, based on TL, to differentiate PGL and Borrmann type IV GC preoperatively. The different-source domain datasets, including the ImageNet dataset, WSIs of the lung, and WSIs of GC, were analyzed. The TLRS based on the pathological GC images had a better performance than TLRS-ImageNet and TLRS-Lung in the internal and two external validation cohorts. Compared with the TLRS-Image, the TLRS had a better performance due to these characteristics. First, the pathological GC images contained pathological information from microscopic observation, and they are in the similar medical images category with the CT images of GC. The extraction features based on the pathological GC images were more specific to the target task, which were more relevant to the task. Moreover, the Wasserstein distance (29), which provides a more meaningful notion of similarity for probability distributions between the source and target domain features, quantifies the correlation between the feature and the model. The Wasserstein distances were calculated for the TLRS and TLRS-ImageNet, which were named the W1 and W2. The results indicated that the W1 was smaller than W2 (0.0433 vs. 0.1157), which manifested that the similarity between the source and target domain features was higher for TLRS.

In addition, compared with the TLRS-ImageNet, the TLRS-Lung demonstrated poor performance in the internal validation cohort (AUC = 0.774), external validation cohort 1 (AUC = 0.787), and external validation cohort 2 (AUC = 0.821) due to the following characteristics. First, the features based on the pathological images of the lung showed a weak correlation with features based on the CT images of GC, which may not be of use to transfer to the target task and even cause negative transformation. Second, although the pathological image of the lung was also a medical image, the results demonstrated that TL does not always result in better performance. Extraction features based on the TLRS-Lung are not easily transferred to the target task (e.g., from the pathological images of the lung to the CT images of GC). The Wasserstein distances were calculated for the TLRS-ImageNet and TLRS-Lung, which were named the W2 and W3. The results indicated that the W2 was smaller than W3 (0.1157 vs. 0.2168), which manifested that the similarity between the source and target domain features was higher for the TLRS-ImageNet.

The results indicated that the source images similar to those in the task might be important in the TL strategy. A similar study showed that TL performance could improve significantly when TL used a source dataset with different images but of the same anatomy (19, 20). Furthermore, in order to verify the effect of transfer learning based on WSIs of GC, some common model were developed and validated: (1) the classical convolutional neural network—VGG16 network (26); (2) the residual neural network (ResNet) (30). Compared to the deep learning without TL, the results showed that the VGG16 and ResNet with WSIs of GC as source domain data also have good performance in different validation cohort (**Table S4**).

**Figure 5:**
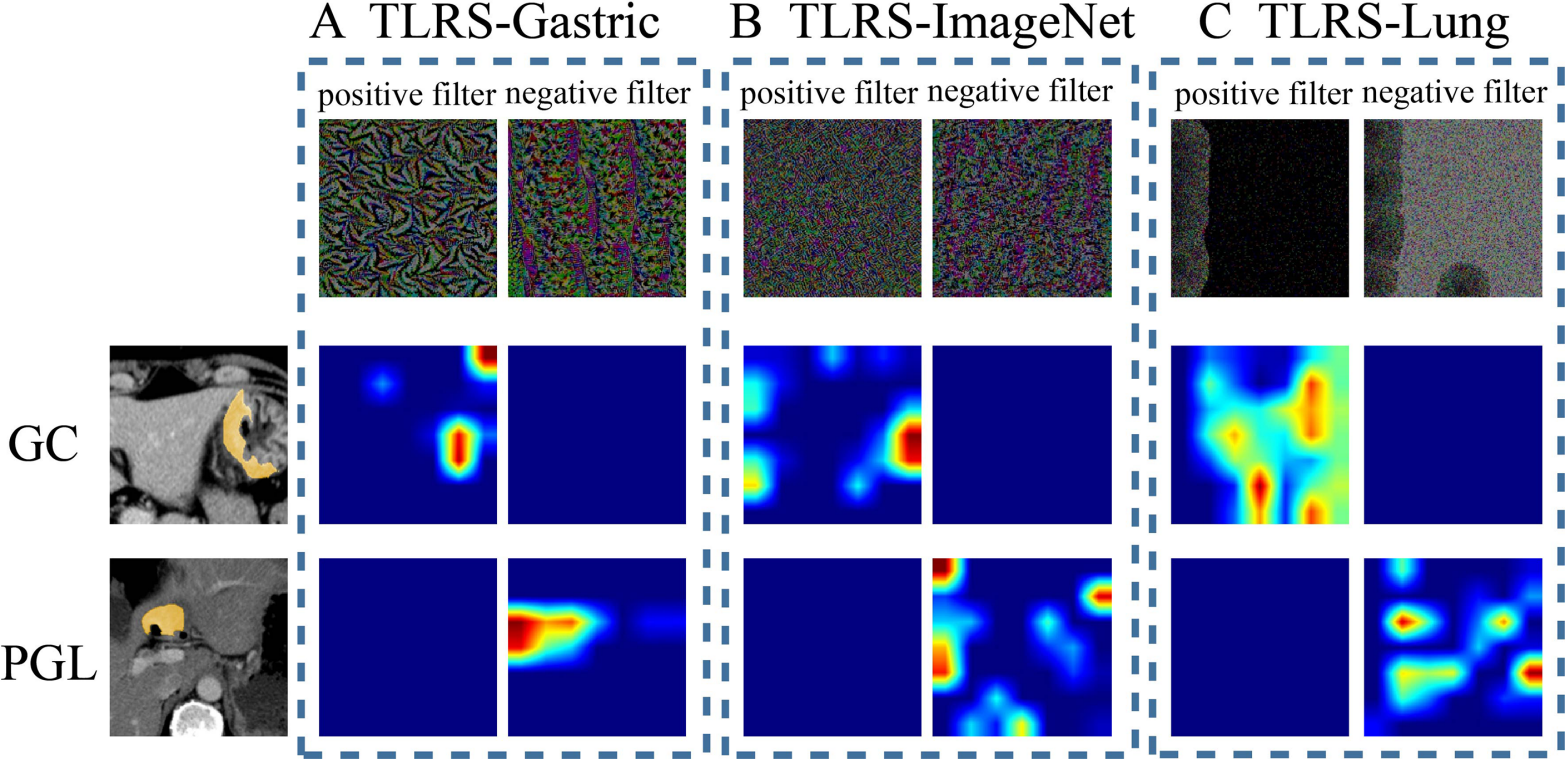
Visualization of two patient samples for the three methods. **(A)** The TLRS-Gastric is based on the WSIs of GC. **(B)** The TLRS-ImageNet is based on the ImageNet dataset. **(C)** The TLRS-Lung is based on the WSIs of the lung. The positive and negative filters for the three methods are in the first row. In the second and third rows, the response heat map of the two patients’ negative and positive transfer learning features was noted. The *red region* represents a larger weight, which shows that the model focuses on the area of the CT image. GC, gastric cancer; PGL, primary gastric lymphoma; TLRS, transfer learning radiomics signature.

In addition, the current study further analyzed the correlation between the features and models, including the TLRS, TLRS-ImageNet, and TLRS-Lung. First, the CNN network visualization algorithm displayed how the network learned the tumor-related information, generating an attention map to identify areas of importance. In the same two patient cases (one patient with Borrman type IV GC and the other with PGL), two filters were visualized (the first column in **Figure 5A**, named the positive and negative filters, respectively) for the three models to explore the association between TL features and lesion images. For the TLRS, the positive filter had strong responses to patients with Borrman type IV GC and weak responses to those with PGL. Similarly, the negative filter had strong responses to patients with PGL and was nearly shut down in patients with Borrman type IV GC (**Figure 5A**). The current study visualized the TLRS-ImageNet model (**Figure 5B**) and TLRS-Lung (**Figure 5C**) models for the same patient cases compared with the TLRS. The results showed that the response degree for the TLRS was stronger than the other two models, and the high-response area was more focused on the lesion.

The TLRN, built by incorporating TLRS, clinical factors, and CT subjective findings, performed better than the other validation sets. The result indicated that the method could mine more image features valuable for diagnosing Borrmann type IV GC and PGL. This study can extract features layer by layer because the TL model consists of multiple layers of autonomous learning units. They contain more lesion information, which is of great value in the diagnosis of this paper. The current method does not need to mark the location of the lesion accurately, but only needs to take CT images as input so that the relationship between the microenvironment information around the lesion and the attached tissues (lymphatic vessels, blood vessels, and so on) can be well evaluated, which can better diagnose Borrmann type IV GC and PGL.

This retrospective study has some limitations. First, the selection of samples was biased when strict inclusion and exclusion criteria were performed, affecting the model training. Second, the TL features were extracted from all tumor lesion slices. However, a single patient’s features were obtained by averaging the features of all lesion images. The current study was based on constructing a two-dimensional slice feature model, and the performance of the three-dimensional features remains to be further studied. Furthermore, the WSI and CT samples are small. Although the current study results show that TLRN has great potential to distinguish PGL and Borrmann type IV GC, adding more samples for further research is necessary. Finally, the TL features were only extracted from arterial and venous phase CT images. However, images from other phases (e.g., plain scan phase), may provide effective clinical guidance.

## Data Availability Statement

The original contributions presented in the study are included in the article/**Supplementary Material**. Further inquiries can be directed to the corresponding authors.

## Ethics Statement

The studies involving human participants were reviewed and approved by the Institutional Review Board of Jiangmen Central Hospital, the First Affiliated Hospital of Sun Yat-sen University and Meizhou People’s Hospital. The ethics committee waived the requirement of written informed consent for participation. Written informed consent for participation was not required for this study in accordance with the national legislation and the institutional requirements.

## Author Contributions

LH, WL, and ZP designed the research. HX, QC, TZ, QK, ZY, XGC, and XFC collected the data. BF, YL, YC, HZ, and TY contributed data analysis tools and performed the analysis, BF also acquired the funding. LH and BF wrote the paper. WL and ZP supervised the study. All authors contributed to the article and approved the submitted version.

## Funding

This work was supported by the National Natural Science Foundation of China (81960324, 61967004 and 61876064), the Guangxi National Natural Science Foundation (2021GXNSFAA075037), the Guangdong Basic and Applied Basic Research Foundation (2019A1515011773), and the Pearl River S&T Nova Program of Guangzhou(201906010043).

## Conflict of Interest

The authors declare that the research was conducted in the absence of any commercial or financial relationships that could be construed as a potential conflict of interest.

## Publisher’s Note

All claims expressed in this article are solely those of the authors and do not necessarily represent those of their affiliated organizations, or those of the publisher, the editors and the reviewers. Any product that may be evaluated in this article, or claim that may be made by its manufacturer, is not guaranteed or endorsed by the publisher.

## Abbreviations

GC, gastric cancer; PGL, primary gastric lymphoma; CT, computed tomography; WSIs, whole slide images; AUC, areas under the curve; CI, confidence interval; DCA, decision curve analysis; ROI, region of interest; ROC, decision curve analysis; PPV, positive predictive value; NPV, negative predictive value; TLRS, transfer learning radiomics signature; TLRPN, transfer learning radiopathomic network; LASSO, least absolute shrinkage and selection operator; TLRN, transfer learning radiomics nomogram; CM, clinical model.
