# Supplementary material for: A Transfer Learning Radiomics Nomogram for Preoperative Prediction of Borrmann Type IV Gastric Cancer From Primary Gastric Lymphoma
Source: Front Oncol. 2022 Jan 11;11:802205. doi: 10.3389/fonc.2021.802205 (PMC8789309; doi:10.3389/fonc.2021.802205)
Supplement: Supplementary file 1 [file DataSheet_1.docx]

**Supplement material**

**Supplementary A1:** **Inclusion and exclusion criteria**

Inclusion criteria: (a) Histologically confirmed Borrmann type IV GC and PGL; (b) Contrast-enhanced abdominal CT examination; (c) No puncture biopsy or chemotherapy or radiotherapy was performed before surgery.

Exclusion criteria included: (a) Substantial motion artifacts in CT images; (b) Poor filling of the stomach with unsatisfactory gastric distention that made it difficult to perform tumor lesions; (c) Patients with significant distant metastases in Borrmann type IV GC, because distant organ metastases and metastatic lymphadenopathy are classically described as heterocyclic ring enhancement, which may be significantly different from PGL; (d) Time intervals between CT and laparoscopy longer than 2 weeks.

**Supplementary A2: CT subjective findings evaluation**

For CT based subjective findings evaluation, a lesion was determined to be cancerous if there was gastric wall thickening or abnormal enhancement of combination of the two compared with the adjacent normal gastric wall. The following subjective findings were evaluated: (a) high enhanced mucosal sign (present or absent), high enhancement in long ribbon shape or patchy shape in the mucosal side around the lesion. (b) high enhanced serosa sign (present or absent), gastric serosa around the lesion with high enhancement in long ribbon shape or patchy shape. (c) nodular or an irregular outer layer of the gastric wall (present or absent), irregular or nodular enhancement around the lesion on gastric serous. (d) perigastirc fat infiltration (present or absent), the outer gastric surface around the lesion exhibiting a lack of a clear perigastric fat plane. The differences in the subjective findings between the two sets were analyzed by the chi-square test.

**Supplementary A3: CT protocol**

The patients underwent contrast-enhanced abdominal CT using Aquilion One-64 (Toshiba Medical Systems, Otawara, Japan). The acquisition parameters are as follows: 120 kVp; tube current, auto; rotation time, 0.5s; detector collimation, 64×0.625 mm or 192×0.625 mm; field of view, 350×350 mm; pitch, 0.656 and 0.7, respectively; matrix, 512×512. The raw data was reconstructed with a 3-mm section thickness for the routine axial CT images.

Following a routine unenhanced scan, a contrast-enhanced CT was performed 25 to 30s (arterial phase) and 60s (venous phase) after an infusion of 1.5 ml/kg the contrast material (Ultravist, Bayer Schering Pharma, Berlin, Germany) was injected at a rate of 3.5 to 4.5 ml/s into the antecubital vein.

**Supplementary A4: The pretreatment of CT images**

The ROI was manually segmented by the radiologists using MATLAB 2018a software (Mathwork Natick, MA, USA) based on the arterial and venous phase image. Since the input of the transfer learning model is rectangular images containing the entire ROI lesions, all slices images of the tumor lesion with each patient were selected as the model input. The slices images were obtained by three steps. First of all, all of the entire ROI tumor lesions were located by radiologist, which were entirely covered by using a rectangle bounding box in Borrmann type IV GC and PGL. Furthermore, the slices of the same rectangular frame were cut consecutively from the tumor lesion images. Finally, each slices of ROI were resized to 224 × 224 size images and standardized using the z-score method. The segmentation process of ROI was showed in **FIGURE S2**.

**Supplementary A5: The transfer learning radiopathomic network training and feature extraction**

The DenseNet121 network was implemented by python keras package (https://github.com/fchollet/kearas) with the TensorFlow library (http://www.tensorflow.org) as the backend.

In this work, the training details of deep learning network involves SGD algorithm to optimize the parameters in the deep learning network iteratively, where the learning rate is 0.0001, decay is 1e-5 and momentum is 0.7. The loss function is categorical crossentropy. The training process converged after 150 epochs, which took almost 5 hours. In addition, our method was implemented in Python 3.7 and performed on a machine with an Intel Xeon Silver 4110 CPU and 64 GB memory. The DL network training was implemented using Keras 2.3.1 and was accelerated on an NVIDIA GeForce GTX 1080Ti GPU (12GB on-board memory). We used a batch size of 32 for model training, which meant 32 training samples were fed into the deep learning network at each iteration.

The transfer learning features were extracted by convolution neural network based on [proposed](javascript:;) TLRPN, where the outputs of the convolutional layer were the transfer learning features. The tumor lesion features were selected based on three steps. First, all of the ROI images were fed to the proposed network. Secondly, the transfer learning features were calculated as the average probability from all slices. Finally, the transfer learning features referred to the output of the convolution layer, and the outputs of transfer learning features were 11264 features.

**Supplementary A6: The software used for statistical test and modeling**

All statistical tests were performed using R3.0.1 (http://www.rproject.org). Transfer learning features extraction and signature generation were applied using a python-based platform developed in-house utilizing the keras package (https://keras.io/) and the opencv package (https://opencv.org/). The statistical analysis was performed using R software 3.0.1 (http://www.rproject.org) with packages including “glmnet”, “pROC”, “rms”, “caret” and “PredictABEL”. P values <0.05 were considered indicative of statistically significant difference.

**Supplementary A7: Transfer learning score formula**

For the transfer learning radiopathomic signature, the transfer learning score (TL-score) calculation formula by LASSO method is as follows:

TL-score = 1.830701 + 0.539942×feature1_AP_conv1_43+0.402843×feature2_AP_conv3_1_x1_60+0.144065×feature3_AP_conv3_blk_200-0.202473×feature4_AP_conv4_blk_309-2.086917×feature5_AP_conv5_blk_bn_110-0.03500×feature6_VP_conv1_15-1.393421×feature7_VP_conv1_18+0.299152×feature8_VP_conv1_38+0.426270×feature9_VP_conv5_9_x2_7-0.044044×feature10_VP_conv5_blk_bn_520

Note: Arterial phase: AP. Venous phase: VP. Conv1_43, conv3_1_x1_60, etc. indicate the filter at this position.

**Supplementary A8: Development of the hand crafted radiomics signature**

The hand crafted radiomics signature (HCRS) was generated with features from CT images, which was building by tumor lesion ROI segmentation, feature extraction, feature selection and model construction based on the Borrmann type IV GC and PGL (the specific process in **FIGURE S5**). There were total of 10402 HCR features were extracted, based on the original image and its corresponding filtered image. And they included the features from the categories of first order statistics (n=78), shape (n=4), Global (n=720), gray-level co-occurrence matrix (GLCM, n=2160), gray-level run-length matrix (GLRM, n=3120), gray-level size-zone matrix (GLSZM, n=3120) and neighborhood gray tone difference matrix (NGTDM, n=1200) [1].

Intraclass correlation coefficients (ICCs) were used to evaluate the reproducibility and stability of the radiomics parameters. Reader 1 performed the segmentation on all of the patients. Reader 2 (with 15 years of experience in abdominal imaging) randomly chose 30 patients from the training cohort and performed tumor segmentation for inter-reader agreement analysis. The radiomics parameters with ICC values greater than 0.75 were considered to be reliable. Then, we used the Mann–Whitney U test to compare the between-group differences of each radiomics feature in the GC and PGL groups. The radiomics features with significant difference and ICC values greater than 0.75 were used for least absolute shrinkage and selection operator (LASSO) logistic regression. The LASSO logistic method was used to select radiomics features with non-zero coefficients as valuable predictors. Finally, the HCRS was built by the linear combination of the selected features.


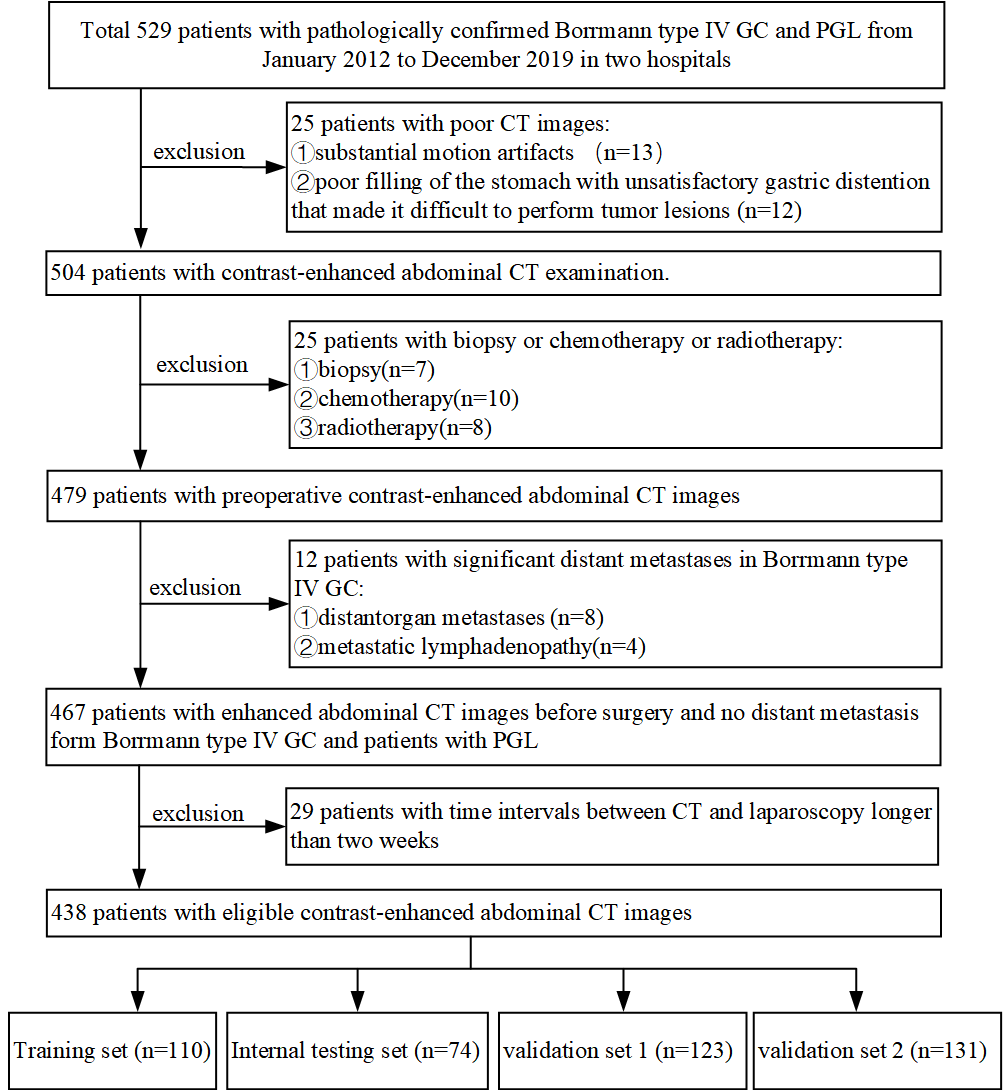
Figure S1. Inclusion and exclusion criteria for patients for training and validation sets.


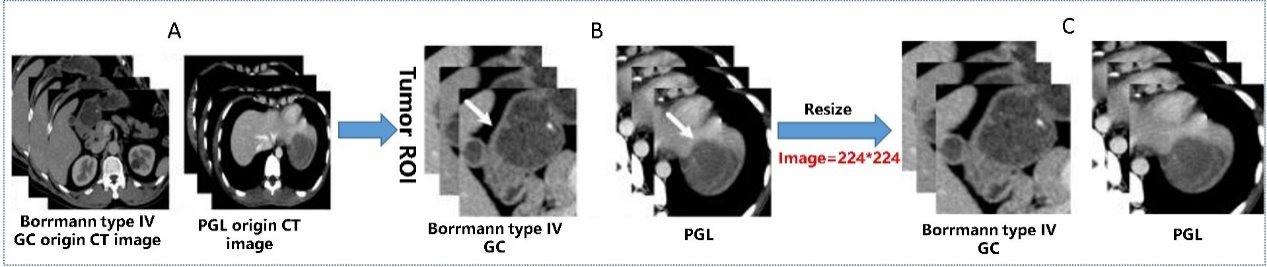


**FIGURE S2**. The segmentation process of ROIs. **A** Original CT images. **B** The tumor ROIs. **C** The ROIs images were resized to 224*224. *GC* gastric cancer, *PGL* primary gastric lymphoma


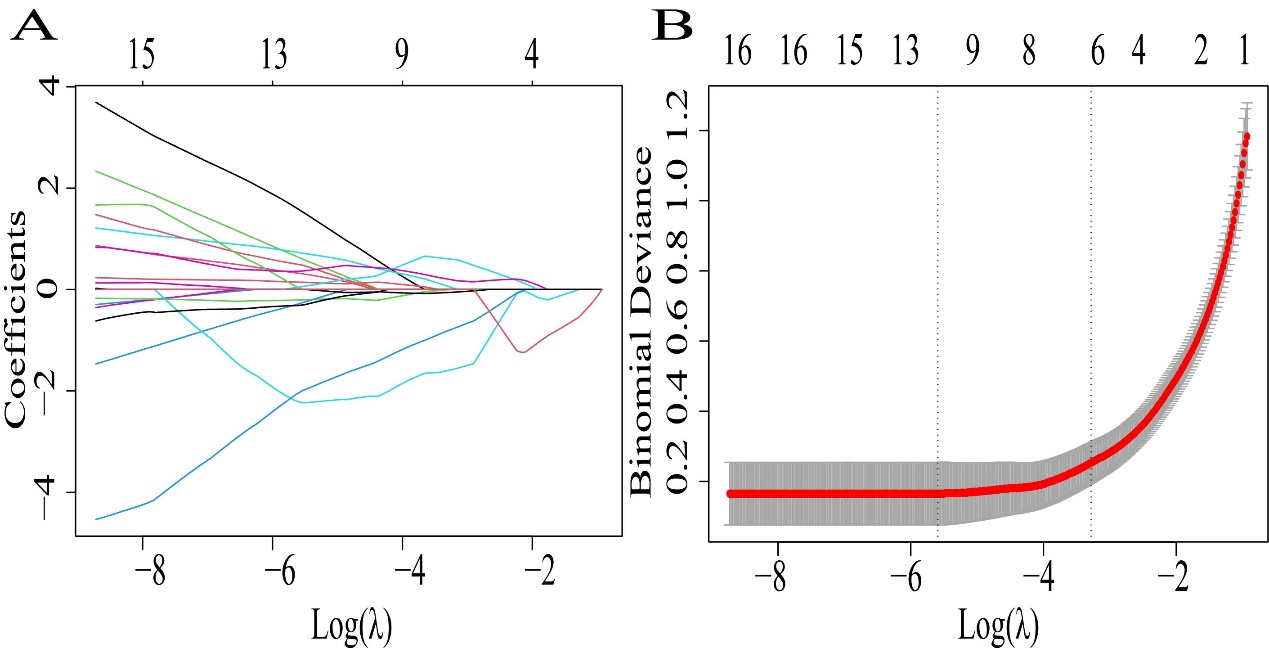


Figure S3. The LASSO regression can reduce the feature dimension by shrinking the coefficients of some features to zero in the training set. The ten-fold cross-validation was used to optimize (**A**), where 10 optimal transfer learning features with non-coefficients are indicated (**B**).


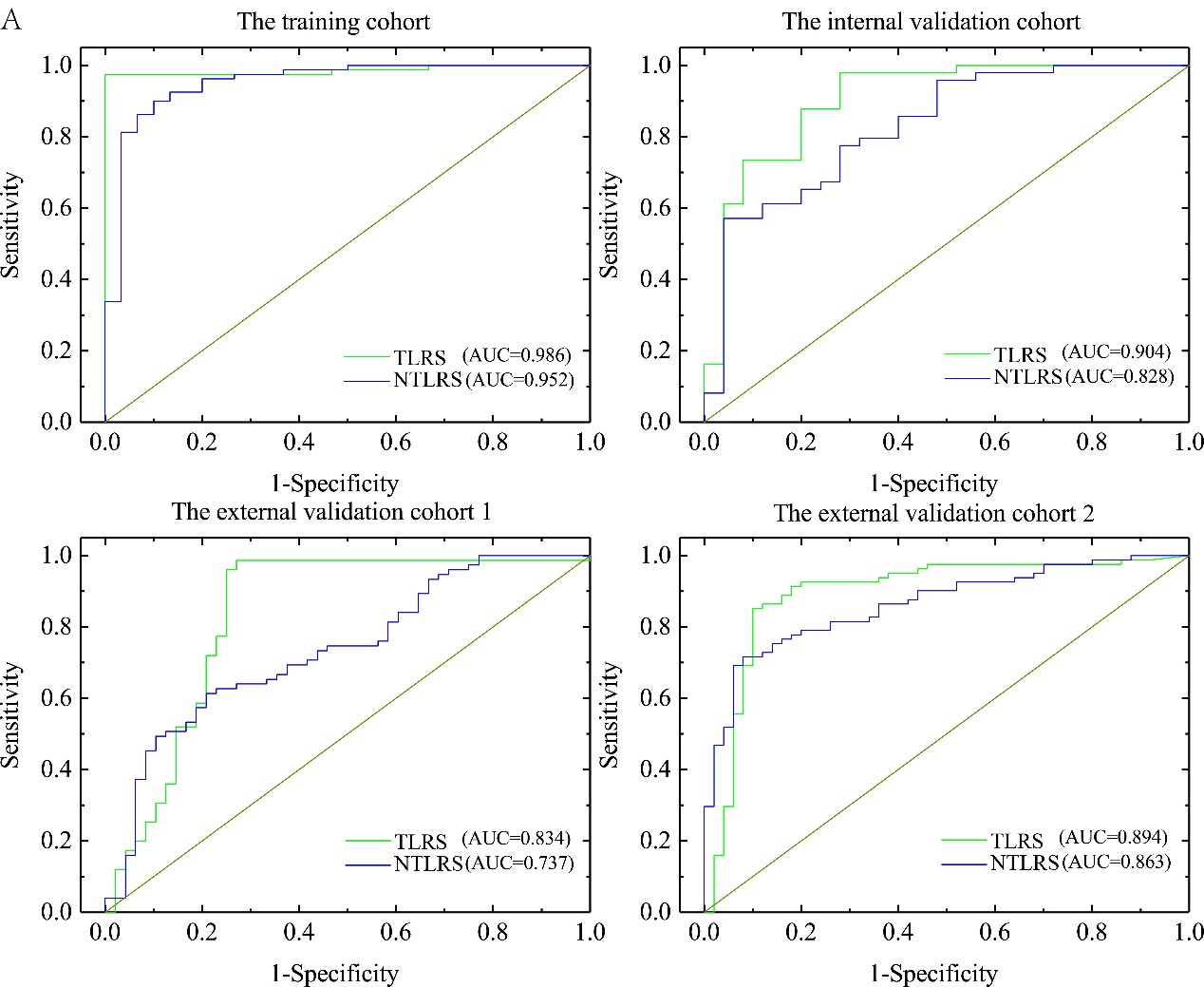


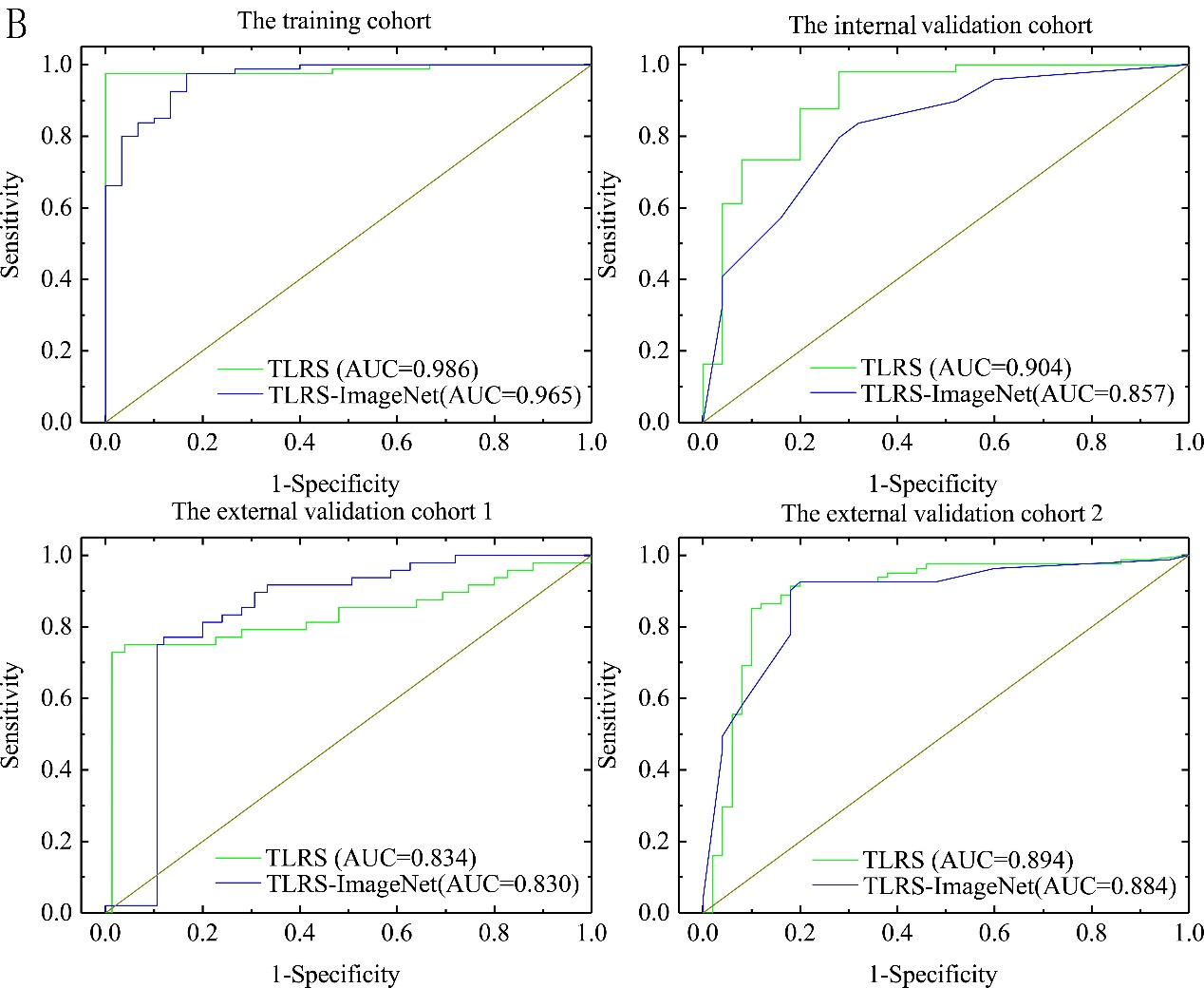


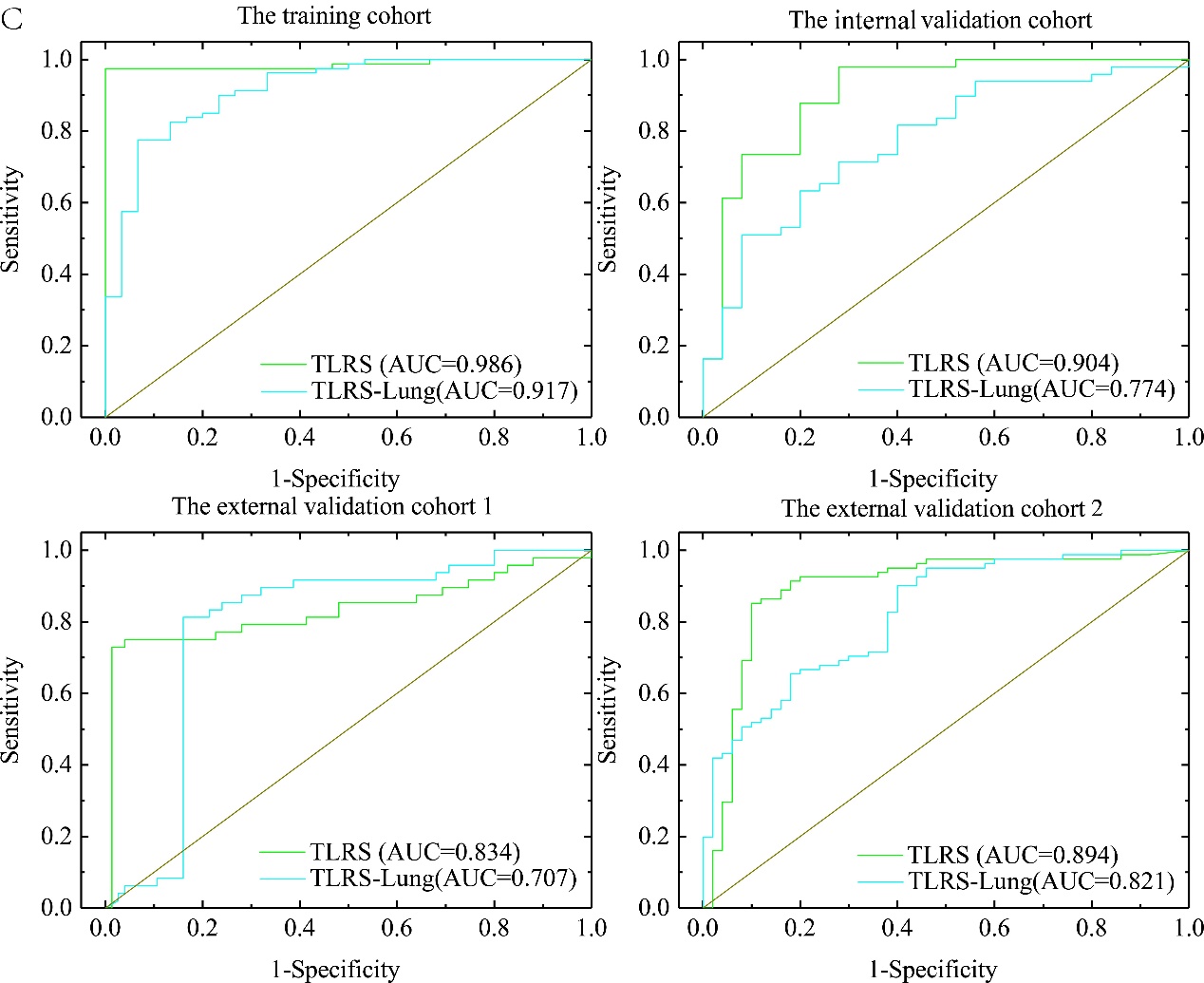


FIGURE S4. The ROC curves of validation cohort. **A** The AUC of transfer learning radiomics signature based on the pathological image of gastric cancer (TLRS) and non-transfer learning radiomice signature (NTLRS). **B** The AUC of transfer learning radiomics signature based on the pathological image of gastric cancer (TLRS) and transfer learning radiomics signature based the ImageNet dataset (TLRS-ImageNet). **C** The AUC of transfer learning radiomics signature based on the pathological image of gastric cancer (TLRS) and transfer learning radiomics signature based on the pathological image of lung (TLRS-Lung).


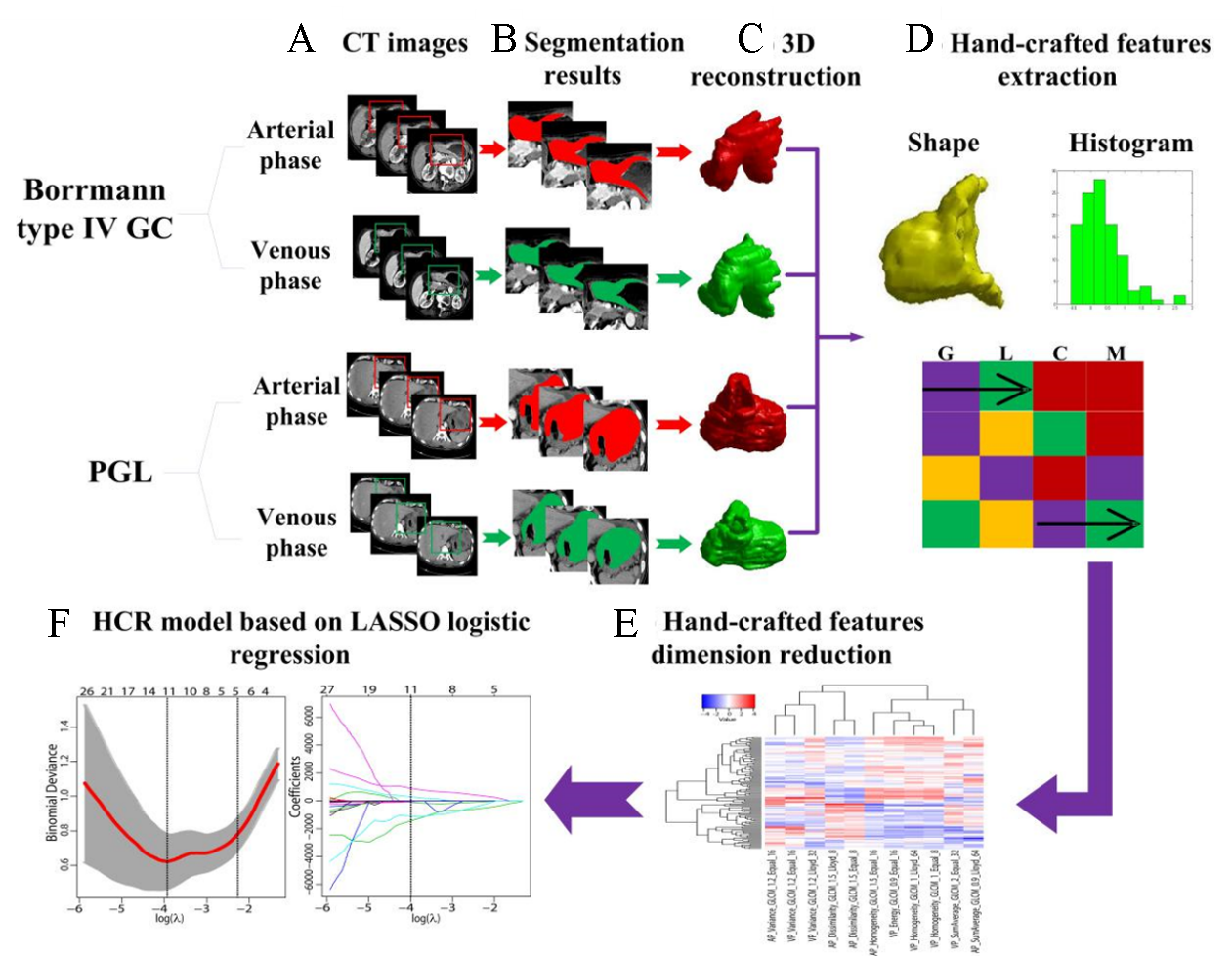


FIGURE S5. The HCRS building flowchart. **A** CT original images. **B** Segmentation results of Borrmann type IV gastric cancer and primary gastric lymphoma. **C** Reconstruction of 2D ROI slices into 3D VOI. **D** Hand-craft features extraction. **E** Hand-craft features dimension reduction. **F** The HCR model based on LASSO logistic regression. *GC* gastric cancer, *PGL* primary gastric lymphoma, and *HCRS*, hand-craft radiomics feature

TABLE S1. Multivariate analysis of risk factors for the clinical model

| Variables |  | Odds Ratio (95%CI) | P-value |
| --- | --- | --- | --- |
| Intercept | 1.599 |  | 0.002* |
| High enhanced serosa sign | -2.285 | 0.102(0.028–0.364) | <0.001* |
| Nodular or an irregular outer layer of the gastric wall | -1.418 | 0.242(0.076–0.769) | 0.016* |
| Perigastric fat infiltration | -1.506 | 0.222(0.069–0.713) | 0.011* |

: regression coefficient; CI: confidence interval. *P<0.05.

TABLE S2. Multivariate analysis of risk factors for the transfer learning radiomics nomogram

| Variables |  | Odds Ratio (95%CI) | P-value |
| --- | --- | --- | --- |
| Intercept | 0.336 |  | 0.657 |
| High enhanced serosa sign | -3.088 | 0.046(0.003, 0.697) | 0.026* |
| TL score | -0.773 | 0.462(0.322,0.661) | <0.001* |

: regression coefficient; CI: confidence interval. *P<0.05. TL, transfer learning.

Table S3. Diagnostic performance of the TLRS, VGG16, ELM and HCRS in the training and validation sets

| **medel** |  | **AUC**  **(95%CI)** | **Sensitive** | **Specificity** | | **Accuracy** | | **PPV** | **NPV** |
| --- | --- | --- | --- | --- | --- | --- | --- | --- | --- |
| **Training cohort** | TLRS | 0.986  (0.942-0.999) | 0.975 | 0.967 | 0.973 | | 0.987 | | 0.936 |
|  | VGG16 | 0.999  (0.998-1.000) | 0.959 | 0.966 | 0.964 | | 0.935 | | 0.979 |
|  | ELM | 0.999  (0.967-1.000) | 0.987 | 1.000 | 0.996 | | 1.000 | | 0.967 |
|  | HCRS | 0.900  (0.829-0.949) | 0.875 | 0.900 | 0.882 | | 0.959 | | 0.730 |
| **Internal validation cohort** | TLRS | 0.904  (0.814-0.961) | 0.980 | 0.720 | 0.892 | | 0.872 | | 0.947 |
|  | VGG16 | 0.671  (0.636-0.704) | 0.613 | 0.729 | 0.446 | | 0.446 | | 0.531 |
|  | ELM | 0.872  (0.774-0.938) | 0.900 | 0.720 | 0.838 | | 0.862 | | 0.783 |
|  | HCRS | 0.837  (0.733-0.912) | 0.735 | 0.962 | 0.813 | | 0.973 | | 0.658 |
| **External validation cohort 1** | TLRS | 0.834  (0.756-0.895) | 0.986 | 0.729 | 0.886 | | 0.850 | | 0.972 |
|  | VGG16 | 0.697  (0.679-0.714) | 0.598 | 0.796 | 0.701 | | 0.730 | | 0.682 |
|  | ELM | 0.756  (0.671-0.829) | 0.693 | 0.792 | 0.732 | | 0.839 | | 0.623 |
|  | HCRS | 0.743  (0.656-0.817) | 0.933 | 0.542 | 0.781 | | 0.761 | | 0.839 |
| **External validation cohort 2** | TLRS | 0.894  (0.828-0.941) | 0.852 | 0.900 | 0.872 | | 0.932 | | 0.790 |
|  | VGG16 | 0.697  (0.679-0.714) | 0.598 | 0.796 | 0.701 | | 0.730 | | 0.682 |
|  | ELM | 0.876  (0.808-0.928) | 0.889 | 0.820 | 0.863 | | 0.889 | | 0.820 |
|  | HCRS | 0.883  (0.815-0.933) | 0.840 | 0.780 | 0.817 | | 0.861 | | 0.750 |

AUC: area under curve. CI: confidence interval. TLRS: transfer learning radiomics signature. VGG: Visual geometry group. ELM: Extreme Learning Machine. HCRS: hand crafted radiomics signature.

Table S4 the performance comparison for transfer learning based on the VGG and ResNet.

| model | | AUC | Sensitivity | Specificity | PPV | NPV |
| --- | --- | --- | --- | --- | --- | --- |
| Internal validation cohort | ResNet-TL | 0.880 | 0.980 | 0.640 | 0.842 | 0.941 |
|  | VGG16-TL | 0.890 | 0.980 | 0.680 | 0.857 | 0.944 |
|  | ResNet-NTL | 0.884 | 0.878 | 0.680 | 0.843 | 0.739 |
|  | VGG16-NTL | 0.863 | 0.959 | 0.640 | 0.839 | 0.889 |
| External validation  cohort 1 | ResNet- TL | 0.691 | 1 | 0 | 0.623 | 1 |
|  | VGG16- TL | 0.700 | 1 | 0.040 | 0.628 | 1 |
|  | ResNet-NTL | 0.510 | 0 | 1 | / | 0.390 |
|  | VGG16-NTL | 0.650 | 0.973 | 0.063 | 0.619 | 0.600 |
| External  validation  cohort 2 | ResNet-TL | 0.831 | 0.933 | 0.146 | 0.631 | 0.583 |
|  | VGG16-TL | 0.834 | 0.973 | 0.042 | 0.613 | 0.500 |
|  | ResNet-NTL | 0.740 | 0 | 1 | / | 0.382 |
|  | VGG16-NTL | 0.680 | 1 | 0.040 | 0.628 | 1 |

TL: transfer learning, NTL: non-transfer learning

**Reference**

[1] Feng B, Chen XM, Chen YH, Lu SL, Liu KF, Li KW, et al. Solitary solid pulmonary nodules: a CT-based deep learning nomogram helps differentiate tuberculosis granulomas from lung adenocarcinomas. Eur Radiol. 2020, 30(12):6497-6507.
